# Persistence in allergen immunotherapy: A longitudinal, prescription data‐based real‐world analysis

**DOI:** 10.1002/clt2.12245

**Published:** 2023-05-06

**Authors:** Oliver Pfaar, Hartmut Richter, Angelika Sager, Christoph Miller, Thomas Müller, Marek Jutel

**Affiliations:** ^1^ Department of Otorhinolaryngology, Head and Neck Surgery Section of Rhinology and Allergy Philipps‐Universität Marburg University Hospital Marburg Marburg Germany; ^2^ Epidemiology IQVIA Frankfurt Germany; ^3^ LETI Pharma GmbH Ismaning Germany; ^4^ Department of Clinical Immunology Wroclaw Medical University Wroclaw Poland; ^5^ ALL‐MED Medical Research Institute Wroclaw Poland

**Keywords:** allergen immunotherapy, persistence, prescription‐based, real‐world analysis

## Abstract

**Introduction:**

Allergic rhinitis (AR) is a widespread disease with increasing prevalence in developed countries. The only treatment that tackles the underlying causes is allergen immunotherapy (AIT). This treatment is performed through two application routes, the subcutaneous immunotherapy (SCIT) or the sublingual immunotherapy (SLIT). However, persistence during the long course of treatment over 3 years is key for the efficacy of this treatment option. The impaired adherence significantly impacts public health resources. The aim of this study was to assess the persistence of AIT for both application routes.

**Methods:**

IQVIA^TM^ LRx was used to identify patients starting AIT between 2009 and 2018 with grass pollen (GP), early flowering tree pollen (EFTP) and house dust mite (HDM) allergens. Patients were classified within each allergen category by AIT groups (subcutaneous depigmented polymerised allergen AIT [dSCIT], other subcutaneous AIT [oSCIT] and SLIT) and age (5‐11 years, 12‐17 years, 18+ years). Furthermore, they were followed up for up to 3 years until the cessation of treatment. Patients, who were still on treatment after 3 years were deemed to be censored. Kaplan‐Meier curves of persistence were generated and compared by log‐rank tests.

**Results:**

The number of patients included in the three allergen categories was 38,717 GP, 23,183 EFTP, and 41,728 HDM AIT. In all allergen categories and for any product group, patient persistence decreased with increasing age class with the difference between 5‐11 years and 12‐17 years greater than between the latter and 18+ years. The percentage of patients completing the first year of AIT was low, particularly for SLIT where 22.2%–27.1% of patients remained persistent after 12 months. The equivalent figures for dSCIT were 52.0%–64.1% and for oSCIT 38.3%–50.3%.

**Conclusion:**

Persistence in AIT in AR was low in this retrospective prescription‐based database and was clearly linked to patient age and application route.

## 1 INTRODUCTION

Allergic rhinitis (AR) is a common medical complaint, particularly in developed countries where prevalence can reach up to 20% of the population.^1^, ^2^ The three main allergen triggers are grass and early flowering tree pollen as well as house dust mites. If uncontrolled, AR is known to be a distinct risk factor for the development of allergic asthma; conversely, up to 80% of asthmatic patients also suffer from AR.^3^ Medical treatment is usually symptomatic,^4^ encompassing for example, antihistamines or anti‐inflammatory nasal steroids.

Allergen immunotherapy (AIT) is the only causal and preventive disease‐modifying therapy available.^5^, ^6^, ^7^ By repeatedly exposing the patient to an allergen, the immune system develops tolerance to allergen exposure. For AIT, several standardized products are available, differentiated by the route of application as subcutaneous immunotherapy (SCIT) or sublingual immunotherapy (SLIT). For SCIT, unmodified allergens or chemically modified allergens (allergoids) are used.^6^ SLIT, on the other hand, is mostly available in the form of unmodified allergens. SCIT is administered by a physician and requires regular visits, whereas SLIT is administered by a patient at home without further supervision. AIT is usually indicated in patients over 5 years of age and can be applied at any age. It is generally accepted that an early introduction to AIT is desirable for this to provide a complete and long‐lasting improvement.^6^

Patient adherence to the treatment regimen and persistence is essential for efficacy of AIT. Patients should be treated for at least 3 years.^4^, ^5^, ^6^, ^7^ Other factors increasing the efficacy of AIT include early initiation after the onset of AR, minor affliction of the lower airways at the time of initiation, young patient age, and a high cumulative AIT dose.^6^ Common problems with AIT include poor adherence and premature termination, which happens more often in SLIT‐treated subjects.^8^, ^9^, ^10^

AIT products show a good safety profile. Efficacy of AIT has been demonstrated in a large number of double‐blind placebo controlled (DBPC) studies, which demonstrate and quantify their effects under standardized clinical conditions. This laid the path for several AIT guidelines.^11^ Numerous trials provide strong evidence for the efficacy of SCIT in pollen and mite allergy‐induced allergic rhinoconjunctivitis in adulthood; however, only a limited number of studies in children and adolescents have been performed.^5^, ^6^ Up to date, the European Medicines Agency's applicable principles on study design and efficacy claims, request the DBPC randomized trial design.^12^, ^13^

Controlled studies feature inherent results bias since patients included are selected according to clearly defined inclusion and exclusion criteria and are instructed and monitored during the course of the study.^14^, ^15^, ^16^ Therefore, the results of randomized controlled trials (RCTs) are only representative of about 5% of the general population, which differs in characteristics such as BMI, comorbidities, or age.^17^ Consideration of the heterogeneity of the population and evidence of reliable real‐world evidence (RWE) studies are therefore essential to assess the efficacy and safety of AIT under real‐world conditions. RWE aims to support RCT evidence by analysing data from daily clinical practice. This approach has been demonstrated to be very helpful, reliable, and complementary to controlled trials in the field of AIT, especially allowing insights into effectiveness in children and adolescents.^9^, ^18^, ^19^

This RWE study aimed to analyse large data on longitudinal prescriptions for SCIT and SLIT over three continuous years of treatment to obtain deeper insight into the persistence rates of patients receiving SCIT and SLIT.

## 2 METHODOLOGY

The database used for the analyses was IQVIA^TM^ LRx which is based on prescription data of statutorily insured patients in Germany. The panel used here covers 60% of the German statutory population. Fully anonymized data is available at the patient level and include demographic information (age, sex) and all information related to prescriptions (product, substance, form, package size, etc.). There is no data available for diagnoses and laboratory tests. Data are available from 2008 onwards and are updated monthly. The last data analysed here was from September 2021.

Prescriptions of AIT (EphMRA ATC: V01A0) in the database were identified and categorized in three allergen groups: grass pollen (GP), early flowering tree pollen (EFTP; birch, alder, hazel), and house dust mites (HDM). Prescriptions from other categories were discarded and multiallergen products were assigned to more than one main group. For each group, the selection timespan was aligned with the main allergen season, starting just after the end of the season when initiation onto AIT was most likely. For HDM the cycle was aligned with the start of the heating period (‘main allergy season’ from September–December). The following selection timespans were used:

- GP: 9/2009–8/2018
- EFTP: 5/2009–4/2018
- HDM: 1/2010–12/2018

Within each group, patients were selected if they satisfied the following criteria:

- Initial prescription in allergen‐specific selection timespan (no AIT in the previous 560 days). The product and prescription date were designated as the index product and the index date respectively.
- Patient aged ≥5 years at index date.

The following three product groups were defined and analysed:

- Depigmented polymerized allergen extract (dSCIT).
- Other subcutaneous AIT (oSCIT).
- Sublingual AIT (SLIT).

For each prescription, the application duration was estimated using the manufacturer's recommendation. For subcutaneous treatments, the duration was based on the total package volume and volume per application considering differential application volumes during updosing. Pre‐seasonally treated SCIT patients were removed from the analyses. Patients were observed until the first of the following occurred:

- Complete cessation of index product AIT.
- Grace period (end to next start of successive prescriptions) in index product AIT exceeding 90 days.
- Switch to non‐index product AIT in the same allergen category.
- End of patient observability

Patients with the first three events were deemed to be nonpersistent, and patients with the last were censored. Kaplan‐Meier curves of persistence were derived for each AIT group over the first three years (1095 days) after the index. The analyses were always stratified by allergen category and product group and were conducted for the overall group as well as by age class at index (children: 5–11 years, adolescents: 12–17 years, adults: 18+ years). Persistence quartiles and mean persistence within the 3 year analysis timespan were calculated and grouped persistence curves compared by log‐rank tests. For comparisons involving more than two groups, a Bonferroni correction was used to maintain the overall *p*‐value at 5% (*p* < 0.05). The software used for the analyses was SAS 9.4.

## 3 RESULTS

**FIGURE 1:**
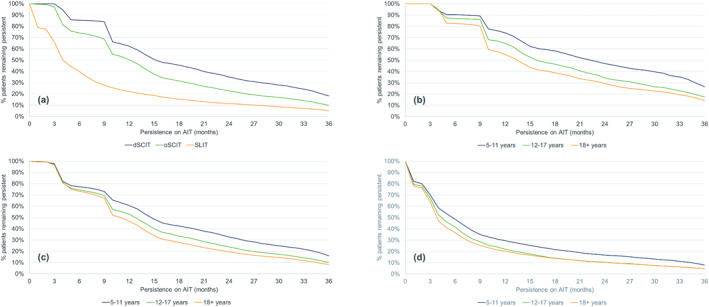
Persistence of patients treated with depigmented polymerized allergen extract (dSCIT), other subcutaneous AIT (oSCIT) and sublingual AIT (SLIT) in the grass pollen category: allergen immunotherapy group comparison (a) and age class comparison for dSCIT (b), oSCIT (c) and SLIT (d).

**FIGURE 2:**
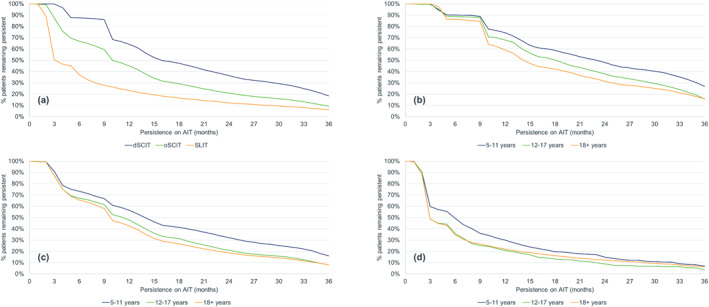
Persistence of patients treated with depigmented polymerized allergen extract (dSCIT), other subcutaneous AIT (oSCIT) and sublingual AIT (SLIT) in the early flowering tree pollen category: allergen immunotherapy group comparison (a) and age class comparisons for dSCIT (b), oSCIT (c) and SLIT (d).

**FIGURE 3:**
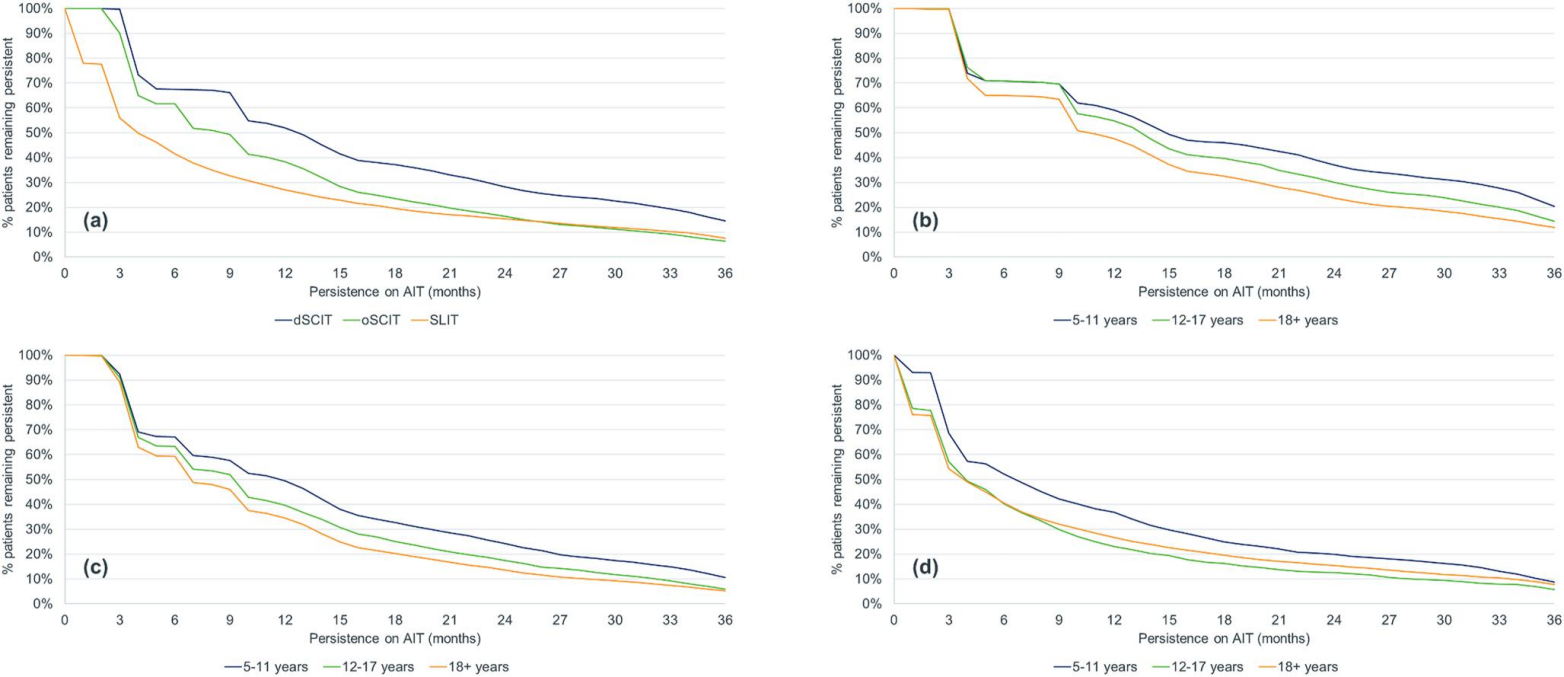
Persistence of patients treated with depigmented polymerized allergen extract (dSCIT), other subcutaneous AIT (oSCIT) and sublingual AIT (SLIT) in the house dust mite category: allergen immunotherapy group comparison (a) and age class comparisons for dSCIT (b), oSCIT (c) and SLIT (d).

A total of 38,717 GP patients (dSCIT: 5532; oSCIT: 13,563; SLIT: 19,622), 23,183 EFTP patients (dSCIT: 4927; oSCIT: 15,141; SLIT: 3115) and 41,728 HDM patients (dSCIT: 12,271; oSCIT: 19,651; SLIT: 9806) were selected for the analyses (Table 1). In all three allergen categories, dSCIT had the highest persistence, followed by oSCIT with SLIT inferior to both (Table 2, Figures 1, 2, 3). Median persistence over all three allergen categories ranged from 389 to 482 days for dSCIT, 258–370 days for oSCIT, and 110–122 days for SLIT.

**TABLE 1:** Statistics for persistence of the overall product groups separated by allergen category.

Allergen	AIT form	N patients	Median persistence (days)	3 years mean persistence (days)	Persistence at 1 year (% patients)	Persistence at 3 years (% patients)
GP	dSCIT	5532	462	582.3	62.2%	18.2%
	oSCIT	13,563	370	463.2	50.3%	9.9%
	SLIT	19,622	122	264.5	22.2%	5.2%
EFTP	dSCIT	4927	482	597.6	64.1%	18.5%
	oSCIT	15,141	300	428.1	45.1%	9.2%
	SLIT	3115	110	277.9	23.2%	6.1%
HDM	dSCIT	12,271	389	492.3	52.0%	14.6%
	oSCIT	19,651	258	369.7	38.3%	6.4%
	SLIT	9806	121	299.8	27.1%	7.6%

Abbreviations: AIT, allergen immunotherapy; dSCIT, subcutaneous depigmented polymerized allergen immunotherapy; EFTP, early flowering tree pollen; GP, grass pollen; HDM, house dust mite; oSCIT, other subcutaneous immunotherapy; SLIT, sublingual immunotherapy.

**TABLE 2:** Statistics for persistence of the product groups separated by allergen category and age class.

Allergen	AIT form	Age class	N patients	Median persistence (days)	3 years mean persistence (days)	Persistence at 1 year (% patients)	Persistence at 3 years (% patients)
GP	dSCIT	5–11 year	1394	684	684.7	74.2%	26.5%
		12–17 years	1287	482	588.3	65.0%	17.8%
		18+ years	2851	409	530.0	55.2%	14.5%
	oSCIT	5–11 years	2303	446	547.1	60.7%	16.2%
		12–17 years	2657	388	474.9	52.9%	10.1%
		18+ years	8603	335	437.2	46.9%	8.2%
	SLIT	5–11 years	3610	174	327.8	19.6%	7.9%
		12–17 years	3608	131	260.7	22.1%	4.7%
		18+ years	12,404	113	247.2	20.2%	4.5%
EFTP	dSCIT	5–11 years	1146	698	689.0	74.4%	27.0%
		12–17 years	788	550	612.0	68.1%	15.6%
		18+ years	2993	434	559.0	59.2%	16.0%
	oSCIT	5–11 years	2097	424	526.5	56.7%	16.1%
		12–17 years	1765	339	435.2	47.9%	8.0%
		18+ years	11,279	280	408.8	42.6%	8.2%
	SLIT	5–11 years	470	180	325.3	30.0%	7.0%
		12–17 years	324	90	252.8	20.7%	3.7%
		18+ years	2321	90	271.9	22.2%	6.2%
HDM	dSCIT	5–11 years	2979	450	565.0	59.3%	20.5%
		12–17 years	2490	411	512.4	54.9%	14.5%
		18+ years	6802	325	453.3	47.8%	12.0%
	oSCIT	5–11 years	3689	360	447.9	49.5%	10.6%
		12–17 years	3766	280	382.4	39.6%	6.0%
		18+ years	12,196	196	342.2	34.5%	5.2%
	SLIT	5–11 years	889	205	371.5	36.9%	8.7%
		12–17 years	1256	119	273.1	23.0%	5.7%
		18+ years	7661	118	295.9	26.7%	7.8%

Abbreviations: AIT, allergen immunotherapy; dSCIT, subcutaneous depigmented polymerized allergen immunotherapy; EFTP, early flowering tree pollen; GP, grass pollen; HDM, house dust mite; oSCIT, other subcutaneous immunotherapy; SLIT, sublingual immunotherapy.

Within the AIT group and based on a combination of parameters (mean and median persistence, persistence at 1 and 3 years), each AIT group performed best in different allergen categories: for dSCIT, the highest persistence was found in the EFTP category, for oSCIT in the GP category and for SLIT in the HDM category (Table 1). For all products and categories, the persistence of most patients was far below the treatment duration of 3 years. The percentage of patients completing the first year of AIT was low, particularly for SLIT where 22.2%–27.1% of patients remained persistent after 12 months. The equivalent figures for dSCIT were 52.0%–64.1% and for oSCIT 38.3%–50.3%. Among patients treated with dSCIT—the group with the highest persistence—14.6%–18.5% completed 3 years of AIT. The values for the two other groups were even lower (oSCIT: 6.4%–9.9%; SLIT: 5.2%–7.6%).

In all allergen categories and for all AIT groups, the highest persistence was recorded for children, followed by adolescents and adults (Table 2, Figures 1b–d, 2b–d, 3b–d). The difference between children and the other two age classes was generally greater than between adolescents and adults and in some cases (e.g. SLIT for EFTP), adolescents had a lower persistence than adults. All comparisons within an allergen category between AIT groups and between age classes within an AIT group were highly significant (*p* < 0.0001) except between the two SCIT groups in the GP category (*p* = 0.1534) and between the age classes for SLIT in the EFTP (significant only for children vs. adolescents) and the HDM (not significant for adolescents vs. adults) categories (Table 3).

**TABLE 3:** Logrank tests for the various comparisons between subgroups separated by allergen category.

Group	Stratum 1	Stratum 2	GP	EFTP	HDM
Overall	dSCIT	oSCIT	0.1534	<0.0001	<0.0001
	dSCIT	SLIT	<0.0001	<0.0001	<0.0001
	oSCIT	SLIT	<0.0001	<0.0001	<0.0001
dSCIT	5–11 years	12–17 years	<0.0001	<0.0001	<0.0001
	5–11 years	18+ years	<0.0001	<0.0001	<0.0001
	12–17 years	18+ years	<0.0001	<0.0001	<0.0001
oSCIT	5–11 year	12–17 years	<0.0001	<0.0001	<0.0001
	5–11 years	18+ years	<0.0001	<0.0001	<0.0001
	12–17 years	18+ years	<0.0001	<0.0001	<0.0001
SLIT	5–11 years	12‐17 years	<0.0001	0.0057	<0.0001
	5–11 years	18+ years	<0.0001	0.1188	0.0002
	12–17 years	18+ years	<0.0001	>0.9999	>0.9999

Abbreviations: dSCIT, subcutaneous depigmented polymerized allergen immunotherapy; EFTP, early flowering tree pollen; GP, grass pollen; HDM, house dust mite; oSCIT, other subcutaneous immunotherapy; SLIT, sublingual immunotherapy.

## 4 DISCUSSION

The expected duration of AIT is 3 years even though a longer treatment duration is possible and many manufacturers' recommendations state that application should be 3–5 years.^6^ In our real‐world study, overall persistence across all allergen categories and AIT groups was low and decreased gradually over the entire analysis timespan, particularly after the first year. There are various possible reasons for treatment cessation, including strong side effects, lack of efficacy, or spontaneous contraindication (e.g. pregnancy in women).^8^ Due to the current study design, it was not possible to define the reasons for the lack of persistence.

Our results are in line with former data from another retrospective analysis based on a Dutch community pharmacy database of 6486 patients receiving SCIT or SLIT.^10^ This analysis also found a low persistence in AIT with only 18% of patients reaching the full treatment course of 3 years (23% in SCIT patients, 7% in SLIT patients). The persistence rates are even lower than those ones reported in previous and rather similar studies based on the same database.^19^, ^20^ For pollen allergens, Vogelberg *et al*.^19^ found SCIT patient persistence after 3 years to be 37.5% (GP) and 35.0% (EFTP) whereas that of various SLIT preparations ranged from 9.6% to 13.4% (GP) and 10.3%–18.2% (EFTP). In the HDM category,^20^ 3‐year persistence reached even higher values: 55.0% for SCIT and 30.3% for SLIT. The HDM values benefitted from a distinctly longer grace period (274 days) but this factor could not have played a part in the pollen analyses where the same duration (90 days) was chosen as in the current analyses. The main difference accounting for the further discrepancies is the fact that in the comparative studies by Vogelberg *et al*.,^19^, ^20^ patients with a single prescription were excluded. This has an impact on the persistence curves raising them, especially in the initial phase, and indicates the major effect that patients discontinuing this treatment after only the first prescription impose on overall persistence.

Taken together, several analytical models confirm poor adherence in general. Indeed, this may have a strong impact on cost‐effectiveness, as results from pharmacoeconomic analyses found that poor persistence rates in AIT increase the cost substantially per Quality‐Adjusted Life Year as recently reported by Di Bona et al.^21^

In addition, distinctions between the age classes were also found with children having consistently the highest persistence, followed by adolescents and finally adults. Medical treatment of children is regularly supervised by their parents so that the higher persistence rates of this age class are expected, and a strong correlation here has been recorded.^22^ With regard to adolescents, it is known that adherence to medicinal therapy is low in this age class in general and often even poorer than that of adults. This phenomenon was not seen here with adolescent persistence generally on a par with or even a little higher than that of adults. The low persistence of adults on AIT seems paradoxical at first sight, implying a less responsible attitude with respect to medical therapy than even that of adolescents. However, it is generally recognized that AIT is more efficacious in young patients. This might negatively impact the persistence of adults. Many reviews could not find a positive correlation between age and implementation of treatment regimen.^22^

Our results clearly demonstrate that SCIT patients performed better than SLIT‐treated individuals. Although it should be considered that oSCIT consisted of a mixture of products, which do not show similar persistence, the differences between dSCIT and oSCIT were substantial in all three allergen categories. It is known that SLIT application leads to a higher rate of milder but discomforting side effects such as oromucosal pruritus or gastric discomfort, especially during the first weeks of therapy.^5^, ^6^, ^23^, ^24^ This might explain the poorer persistence of SLIT patients. Furthermore, the results have shown that a drop of adherence is seen earlier in the SLIT group than in the SCIT groups. Since the reach of the updosing packaging units for therapy start of SLIT products (e. g. 30 tablets for 1 month) is much shorter than the corresponding packaging units of SCIT products, missing follow‐up prescriptions are noticed earlier in the SLIT group. It has been also observed in several previous studies that SLIT patients show lower persistence than those on SCIT but the distinction between different SCIT forms has so far not been investigated in detail.^19^, ^20^, ^25^ In the group of other SCIT, all SCIT products except for depigmented polymerized allergoids were included. It means, that this group contains modified allergoids as well as native allergen extracts with different dosage regiments and different allergen content and therefore different efficacy and rates of side effects. On the other side, dSCIT contains one defined product. This might explain the differences between the two SCIT groups. Poor adherence to the treatment regimen and persistence has been identified as a general contraindication for AIT as it is essential for successful therapy.^4^, ^5^, ^6^ It still remains a critical unmet need to improve adherence and persistence.^6^

RWE studies are increasingly expected to play an important role in the future and to complement RCTs in the assessment of safety and effectiveness in order to provide a comprehensive picture of the ‘real world’.^17^, ^26^, ^27^, ^28^ Paoletti *et al*.^17^ recently published a systematic review of observational studies of AIT calling for appraisal of the quality and importance of RWE in AIT. They underline the need for further collaborations to standardize the methodology for RWE studies. In this regard, even regulatory bodies such as the FDA are convinced of the added value of RWE studies and are working on the framework of the ‘RWE Programme’.^29^, ^30^ Our RWE study may serve as a model for further analyses aimed to improve the clinical efficacy of AIT as the only existing disease modifier in the future.

## 5 LIMITATIONS

The main limitation of the database with respect to the current investigation is the lack of prescription posology information which is crucial in determining the duration of a prescription. Since all other prescription information (allergen, starter/maintenance form, package size, count, etc.) was available, it was possible to make reliable estimates using the recommended application regimes of the manufacturers. This was particularly the case for SLIT where these regimes are rigidly fixed. Any remaining dosage uncertainties were minimized by the fact that prescription overlap (signifying overuse) was ignored and short pauses between prescriptions (signifying underuse) were tied over by means of the grace period.

## 6 CONCLUSIONS

Persistence on AIT therapy in the three allergen categories assessed was distinctly higher for SCIT than for SLIT. Children showed consistently higher persistence than both adolescents and adults. Nevertheless, the persistence of all groups analysed under real‐world conditions was low and it is likely that treatment intensity in a substantial proportion of patients was too low to exert efficacy. It would be desirable to elucidate the reasons for low AIT persistence to take proper measures wherever possible. Improving strategies for optimizing adherence to AIT poses one of the most relevant aims for future developments in AIT.

## AUTHOR CONTRIBUTION

Oliver Pfaar and Angelika Sager proposed the study, analysed the data and wrote the paper in collaboration with Hartmut Richter (IQVIA). Hartmut Richter performed the analysis and wrote the first draft of the paper in collaboration with AS and Oliver Pfaar. Christoph Miller, Thomas Müller and Marek Jutel participated in the discussion of the results and contributed to the paper. All authors agreed to its publication.

## CONFLICT OF INTEREST STATEMENT

Oliver Pfaar reports personal fees from IQVIA Commercial during the conduct of this trial. He reports grants and/or personal fees from ALK‐Abelló, Allergopharma, Stallergenes Greer, HAL Allergy Holding B.V./HAL Allergie GmbH, Bencard Allergie GmbH/Allergy Therapeutics, Lofarma, ASIT Biotech Tools S.A., Laboratorios LETI/LETI Pharma, GlaxoSmithKline, ROXALL Medizin, Novartis, Sanofi‐Aventis and Sanofi‐Genzyme, Med Update Europe GmbH, streamedup! GmbH, Pohl‐Boskamp, Inmunotek S.L., John Wiley and Sons, AS, Paul‐Martini‐Stiftung (PMS), Regeneron Pharmaceuticals Inc., RG Aerztefortbildung, Institut für Disease Management, Springer GmbH, AstraZeneca, IQVIA Commercial, Ingress Health, Wort&Bild Verlag, Verlag ME, Procter&Gamble, ALTAMIRA, Meinhardt Congress GmbH, Deutsche Forschungsgemeinschaft, Thieme, Deutsche AllergieLiga e.V., AeDA, Alfried‐Krupp Krankenhaus, Red Maple Trials Inc., Technical University Dresden, all outside the submitted work; and he is member of EAACI Excom, member of ext. Board of directors DGAKI; coordinator, main‐ or co‐author of different position papers and guidelines in rhinology, allergology and allergen‐immunotherapy. Hartmut Richter is employee of IQVIA, Frankfurt/Main, Germany. Angelika Sager is employee of the sponsor company. Christoph Miller is employee of the sponsor company. Thomas Müller is employee of the sponsor company. Marek Jutel reports personal fees from Allergopharma, ALK, Stallergenes, Hal, Allergy Therapeutics, Leti, GSK, Novartis, Genentec, TEVA, TAKEDA, Chiesi, Shire, Janssen, Celltrion, Sanofi, Regeneron, Roche all outside of the submitted work.
